# An Application of Wastewater Treatment in a Cold Environment and Stable Lipase Production of Antarctic Basidiomycetous Yeast *Mrakia blollopis*

**DOI:** 10.1371/journal.pone.0059376

**Published:** 2013-03-14

**Authors:** Masaharu Tsuji, Yuji Yokota, Kodai Shimohara, Sakae Kudoh, Tamotsu Hoshino

**Affiliations:** 1 Biomass Refinery Research Center (BRRC), National Institute of Advanced Industrial Science and Technology (AIST), Kagamiyama, Higashihiroshima, Hiroshima, Japan; 2 Bio-Production Research Institute, National Institute of Advanced industrial Science and Technology (AIST), Tsukisamu-higashi, Toyohira-ku, Sapporo, Hokkaido, Japan; 3 Hokkaido High-Technology College (HHT), Megunino-kita, Eniwa, Hokkaido, Japan; 4 National Institute of Polar Research (NIPR), Midori-cho, Tachikawa, Tokyo, Japan; 5 Graduate School of Life Science, Hokkaido University, Kita-ku, Sapporo, Hokkaido, Japan; Louisiana State University, United States of America

## Abstract

Milk fat curdle in sewage is one of the refractory materials for active sludge treatment under low temperature conditions. For the purpose of solving this problem by using a bio-remediation agent, we screened Antarctic yeasts and isolated SK-4 strain from algal mat of sediments of Naga-ike, a lake in Skarvsnes, East Antarctica. The yeast strain showed high nucleotide sequence homologies (>99.6%) to *Mrakia blollopis* CBS8921^T^ in ITS and D1/D2 sequences and had two unique characteristics when applied on an active sludge; i.e., it showed a potential to use various carbon sources and to grow under vitamin-free conditions. Indeed, it showed a biochemical oxygen demand (BOD) removal rate that was 1.25-fold higher than that of the control. We considered that the improved BOD removal rate by applying SK-4 strain was based on its lipase activity and characteristics. Finally, we purified the lipase from SK-4 and found that the enzyme was quite stable under wide ranges of temperatures and pH, even in the presence of various metal ions and organic solvents. SK-4, therefore, is a promising bio-remediation agent for cleaning up unwanted milk fat curdles from dairy milk wastewater under low temperature conditions.

## Introduction

Drainage from dairy parlors and milk factories produced in the process of cleaning transport pipes and milking tanks pollute rivers and groundwater with detergents, bactericides, mucus and milk fat are contaminating rivers and underground water [1]. In low temperature conditions, the wastewater is treated by bio-filters [2] and a reed bed system [3], [4]. However, the system is not used widely because of the high running cost and the necessity of a large space. Instead, an activated sludge system is now widely used for industrial treatment of dairy parlor wastewater [5] due to its advantages in maintenance and running cost. However, there is a problem in this system of low temperature conditions in winter having adverse effects on microbial functions.

The use of microorganisms living in polar regions for the purpose of removing nitrogen and phosphorus compounds from wastewater under low temperature conditions has been reported by Chevalier et al. [6] and Hirayama-katayama et al. [7], but it has not yet been applied for milk fat.

In our previous work, we examined 305 isolates of fungi including eight Ascomycetous and six Basidiomycetous species collected from Antarctica and found that they included fungi of the genus *Mrakia*, in psychrophilic Basidiomycetous yeast, suggesting that *Mrakia* is a major mycoflora highly adapted to the Antarctic environment (Fujiu, 2010; master's thesis in Graduate School of Science, Hokkaido University). *Mrakia* spp. and *Mrakiella* spp. are also common fungal species frequently found in cold climate areas such as Arctic, Siberia, Central Russia, the Alps and Antarctica [8]–[10]. Therefore, we screened our *Mrakia* isolates for their ability to decompose milk fat under low temperature conditions and evaluated their potential for application to an active sludge system in a region with a cold climate. The results showed that 56 *Mrakia* spp. exhibited a clear zone according to fat decomposition. Antarctic yeast strain SK-4 had physiological characteristics similar to those of *Mrakia blollopis* [11].

Here we report that activated sludge containing yeast strain SK-4 has the potential to remove milk fat BOD_5_. We also describe identification of yeast strain SK-4 and the purification and characterization of the lipase, considered as a major enzyme to degrade milk fat in wastewater.

## Materials and Methods

### Ethics statement

All necessary permits were obtained for the described field studies. Permission required for field studies was obtained from the Ministry of the Environment of Japan. Sample collection in Antarctica was performed with the permission of the Ministry of the Environment of Japan.

### Sample isolation

Algal mat samples were collected from sediments of Naga-ike, a lake in Skarvsnes, located near Syowa station, East Antarctica. The isolate was inoculated on potato dextrose agar (PDA) (Difco^TM^, BD Japan, Tokyo, Japan) at 4°C for 1 week. Yeast strain SK-4 was selectively picked for isolation on the basis of its morphology. Yeast strain SK-4 was maintained on PDA plates at 4°C and long-term storage was performed in 40% (w/v) glycerol at −80°C.

### Phylogenetic analysis

Phylogenetic analysis was done by sequencing the ITS region including 5.8S rRNA and D1/D2 domain of 26S rRNA. Cells were harvested from 2-weeks-old cultures. DNA was extracted with an ISOPLANT II kit (Wako Pure Chemical Industries, Osaka, Japan) according to the manufacturer's protocol. Extracted DNA was amplified by PCR using KOD-plus DNA polymerase (TOYOBO, Osaka, Japan). The ITS region was amplified by using the following primers: ITS1F (5′-GTA ACA AGG TTT CCG T) and ITS4 (5′-TCC TCC GCT TAT TGA TAT GC). The D1/D2 domain was amplified using the following primers: NL1 (5′-GCA TAT CAA TAA GCG GAG GAA AAG) and NL4 (5′-GGT CCG TGT TTC AAG ACG G). Sequences were obtained with an ABI prism 3100 Sequencer (Applied Biosystems, Life Technologies Japan, Tokyo, Japan) using an ABI standard protocol. The ITS region and D1/D2 domain sequences of yeast strain SK-4 are deposited in DNA Data Bank of Japan (BBDJ) (Accession numbers AB630315 and AB691134). Alignment was made using CLUSTAL W (http://clustalw.ddbj.nig.ac.jp/) and corrected manually. Phylogenetic analysis was performed using MEGA software version 4.0 [12] with neighbor-joining analysis of the ITS region containing 5.8S rRNA and maximum parsimony analysis of the D1/D2 domain of 26S rRNA. Bootstrap analysis (1000 replicates) was performed using a full heuristic search.

### Physiological characterization

Assimilation of carbon was performed at 15°C on modified Czapek-Dox agar composed by 6.7 g/L of yeast nitrogen base without amino acids (Difco^TM^, BD Japan, Tokyo, Japan), 2.0 g/L of sodium nitrate (Wako Pure Chemical Industries, Osaka, Japan), 30 g/L of carbon source and 15.0 g/L of Agar (Difco^TM^, BD Japan, Tokyo, Japan). Assimilation of nitrogen and other physiological tests were carried out according to the protocols described by Yarrow [13]. All tests were performed at 15°C after 2 and 4 weeks of inoculation.

### Preparation of active sludge and measurement of biochemical oxygen demand

Activated sludge (AS) was cultivated at room temperature with aeration by using cow's milk as the substrate. After one month, the sludge was divided into two parts. One part of the activated sludge was mixed with *M. blollopis* SK-4 (1.4 g/L, dry weight), and the other part was used as a control. Separated activated sludge was prepared with Mixed Liquor Suspended Solids (MLSS, 3000 mg/L) and cow's milk at 10°C with aeration. One week later, prepared activate sludge was added to cow's milk, and biochemical oxygen demand (BOD_5_) of waste-treated water was measured after 24 hours. BOD_5_ assay was carried out using a coulometer (Ohkura Electric, Saitama, Japan).

### Inoculum

*M. blollopis* SK-4 was grown in YPD liquid medium (1% yeast extract, 2% peptone, and 2% glucose) at 15°C for 96 hours at 120 rpm. After 96 hours, *M. blollopis* SK-4 was collected by centrifugation at 3500×g for 15 min at 4°C. The pellet was transferred to fresh cream liquid medium (0.5% peptone, 0.5% NaCl, 5% fresh cream, pH 7.0) and incubated at 10°C for 14 days at 90 rpm. The resulting culture was used as inoculum.

### Lipase production medium

Lipase production medium was composed of 0.2% KH_2_PO_4_, 0.29% Na_2_PO_4_, 0.02% NH_4_Cl, 0.04% CaCl_2_, 0.001% FeCl_3_, 0.5% yeast extract, and 1% Tween 80. The yeast was cultivated at 10°C for 324 hours at 90 rpm. One mL samples were collected every 24 h and centrifuged at 4°C for 10 min at 20000×g, and then lipase activity was measured.

### Assay of lipase activity

Lipase activity was measured by a colorimetric method using p-nitrophenyl-palmitate as a substrate [14]. Forty mL of 50 mM sodium phosphate buffer (pH 7.0) containing 50 mg gum arabic and 0.2 g TritonX-100 was mixed with 3 mL 2-propanol containing 1 mM p-nitrophenyl-palmitate. Eight hundred μL of prepared substrate was added to 200 μL of enzyme solution. The enzyme reaction was carried out at 30°C for 30 min. The released p-nitrophenol was measured at A_410_. One unit of lipase activity was defined as the activity required to release 1 μmol of free fatty acids per minute at 30°C.

### Measurement of protein concentration

Protein concentration was measured by BCA protein assay reagent (Thermo Fisher Scientific, Waltham, MA, USA) according to the manufacturer's instructions using bovine serum albumin as a standard.

### Purification of lipase

*M. blollopis* SK-4 lipase was purified by ultrafiltration and Toyopearl-butyl 650 M (Tosho, Tokyo, Japan) hydrophobic interaction chromatography. Four hundred mL of lipase production liquid medium was centrifuged at 4°C for 15 min at 3000×g. The supernatant was filtered through a 0.45-μm of membrane filter (Advantec, Tokyo, Japan). The filtered medium was concentrated by ultrafiltration using an ultracel YM-30 membrane (Millipore, Billerica, MA, USA). The concentrated sample was adsorbed to a Toyopearl butyl 650 M column (2.5×20 cm) containing 1 M sodium chloride and eluted with a linear gradient from 750 mM to 100 mM sodium chloride in 20 mM Tris-HCl buffer (pH 8.5) at a flow rate of 60 mL/h. Fractions of high lipase activity was pooled and concentrated and then stored at 4°C until use. Protein molecular weight was estimated by SDS-PAGE according to Laemmli [15] and stained with CBB R-250. Precision plus protein unstained standards (Bio-Rad Laboratories Japan, Tokyo, Japan) were used as protein molecular weight makers.

### Characterization of lipase

Substrate specificity was determined by using substrate as different p-nitrophenyl esters (C_4_–C_18_). For determining the effects of metal ions and EDTA on lipase activity, residual lipase activity assays were carried out under standard assay conditions with final concentrations of 1 mM of various bivalent metal ions and EDTA. Lipase activity assay in the absence of metal ions and EDTA was carried out as a control. Optimum pH was measured at 30°C for 30 min and determined at various pH values of 50 mM buffer as follows: sodium citrate (pH 3.0–5.0), sodium phosphate (pH 6.0, 7.0 and 8.0), Tris-HCl (pH 7.5 and 8.5), glycine-NaOH (pH 9.0) and sodium carbonate (pH 9.3, 9.5 and 10.0).

Optimum temperature was measured in 50 mM sodium phosphate buffer (pH 7.0) for 30 min. To determine the pH stability of lipase, the enzyme was preincubated in various buffers for 15 h at 30°C and then adjusted to pH 8.5. The residual enzyme activity was measured by using p-nitrophenyl-palmitate as a substrate at 65°C for 30 min. For determining thermo-stability, lipase was preincubated for 30 min at different temperatures and the residual activity was measured at 65°C for 30 min in 50 mM Tris-HCl (pH 8.5). Effects of organic solvents on lipase activity were determined at 65°C for 30 min in 50 mM Tris-HCl (pH 8.5) containing various organic solvents at final concentration of 5% (v/v). Lipase activity assay in the absence of organic solvents was carried out as a control.

## Results

### Phylogenetic analysis

**Figure 1:**
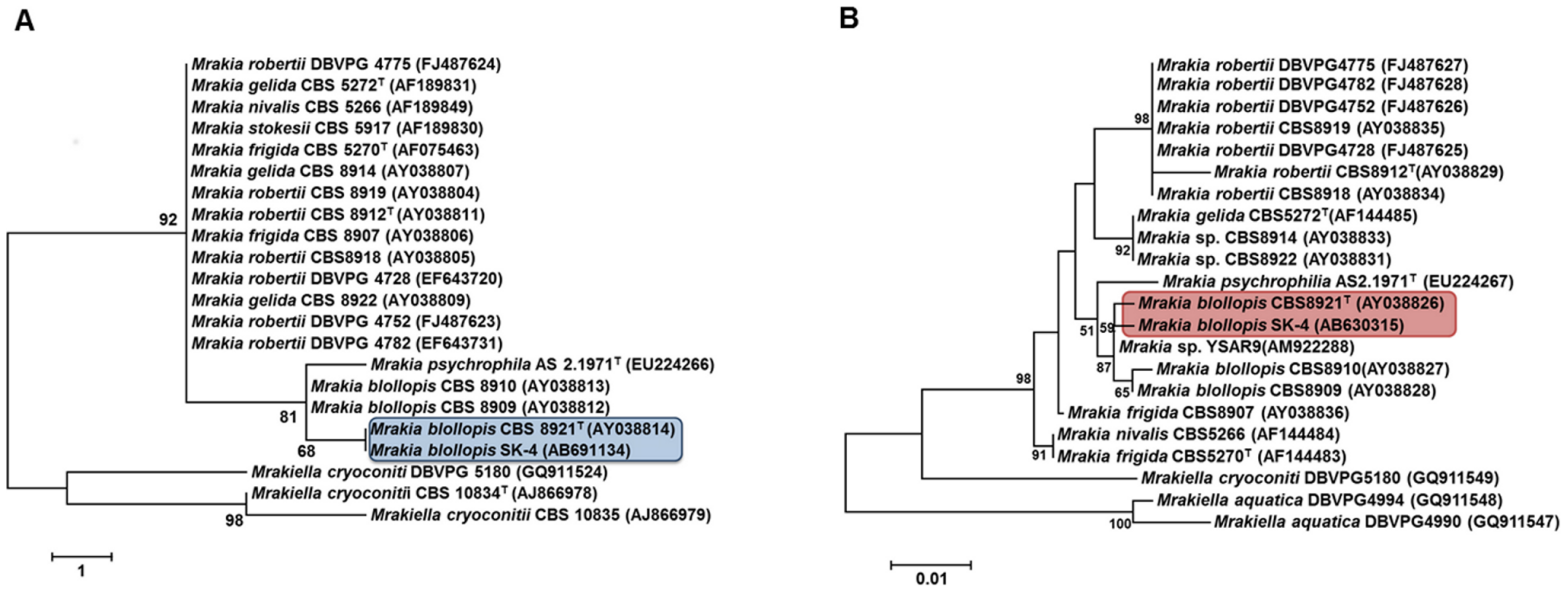
Phylogenetic tree of *Mrakia blollopis* SK-4 and related species. (A) Maximum parsimony analysis of the D1/D2 domain of the 26 rRNA gene sequence. Bootstrap percentages from 1000 replications are shown on the branches. *Mrakiella cryoconiti* CBS 10834^T^ and *Mrakiella cryoconiti* CBS 10835 were used as an out group. (B) Neighbor-joining tree of the ITS region containing the 5.8 S rRNA gene sequence. Bootstrap percentages from 1000 replications are shown on the branches. *Mrakiella aquatica* DBVPG4994 and *Mrakiella aquatica* DBVPG4990 were used as an out group.

As a result of phylogenetic analysis of the ITS region and D1/D2 domain, yeast strain SK-4 was grouped with the clade of *Mrakia blollopis* CBS 8921^T^ (Fig. 1 A and B). By comparison of the ITS region sequence containing 5.8 S rRNA, yeast strain SK-4 showed high homologies (>99.6%) with *M. blollopis* CBS8921^T^. The D1/D2 domain sequence showed no variation with *M. blollopis* CBS 8921^T^.

### Physiological characterization

Results of assimilation of carbon compounds and other physiological tests of *M. blollopis* SK-4 are shown in Table 1 with its type strain and related species. Test data for *M. blollopis* SK-4 are compared with those for *M. blollopis* CBS8921^T^ [8], *M. psychrophila* AS2.1971^T^ [16], and *M. frigida* CBS5270^T^ [17]. Maximum growth temperature of *M. blollopis* SK-4 was 22°C. Maximum growth temperatures of other related species were lower than 20°C. *M. blollopis* SK-4 differed from the other strains in substrate utilization as well. The strain could thrive well on lactose, D-arabinose, and inositol medium. Unlike other strains, this strain also grew on vitamin-free medium. A comparison of fermentabilities showed that *M. blollopis* SK-4 could ferment typical sugars such as glucose, sucrose, galactose, maltose, lactose, raffinose, trehalose and melibiose, while other related species were not able to strongly ferment such as various sugars (Table 1).

**Table 1:** Comparison of physiological characteristics of *Mrakia blollopis* SK-4 and other *Mrakia* species.

Characteristic	*M. blollopis* SK-4	*M. blollopis* CBS8921^T^	*M. psychrophhila* AS2.1971*^T^*	*M. frigida* CBS5270^T^
Maximum growth temperature	22°C	20°C	18°C	17°C
Assimilation of				
Lactose	+	W	+	V
Inositol	+	w/+	+	V
D-arabinose	+	w/−	+	V
Ethanol	w/−	+	+	+
Growth on 50% glucose	w/−	−	+	−
Growth on vitamin-free medium	+	W	+	−
Fermentation of				
Galactose	+	−	−	W
Lactose	+	−	−	−
Raffinose	+	−	−	W
Maltose	+	−	−	W

Main physiology test results for characteristics of *M. blollopis* SK-4 and related species are shown. Physiological data were taken from Fell et al. (1969), Xin and Zhou (2007), Thomas-Hall et al. (2010) and this study. +, positive; w, weak; −, negative; v, variable; nd, no data.

### Assessment of milk fat decomposition in model wastewater

**Figure 2:**
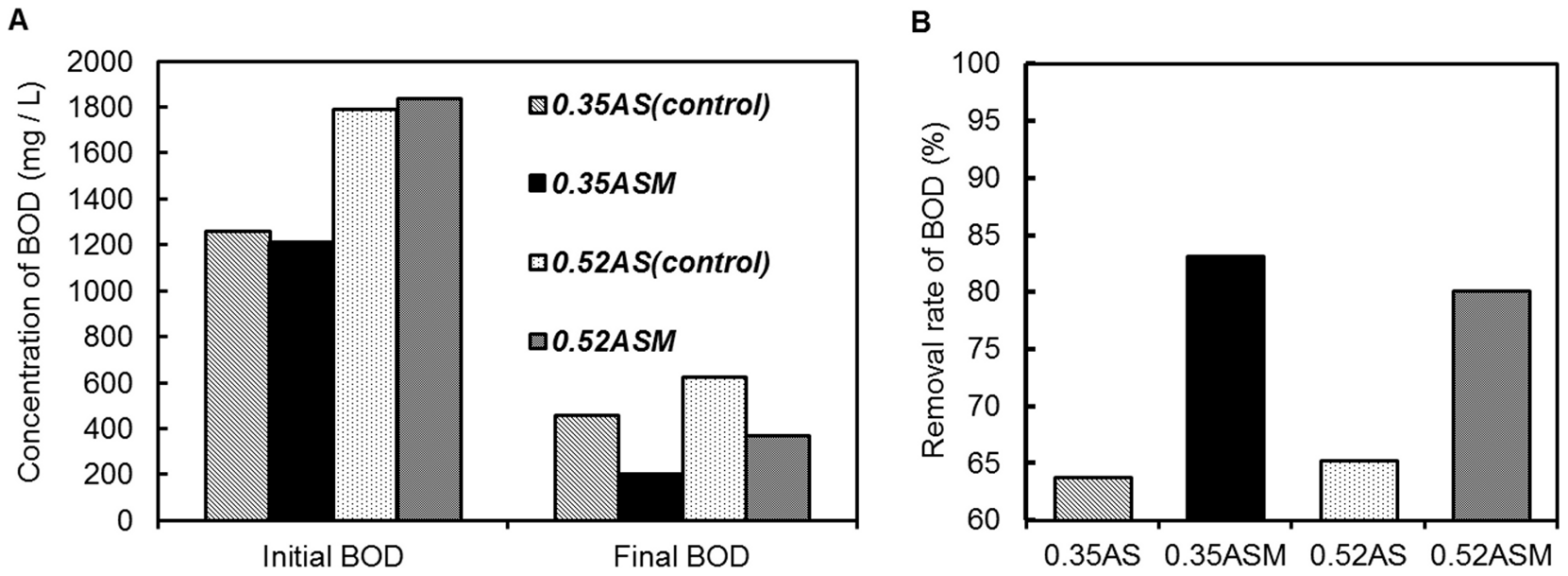
Performance of activated sludge system treating model milking parlor wastewater. (A) Results of measurement of initial BOD and final BOD. (B) Effect of BOD removal rate by ASM treatment. (0.35AS) for 0.35 BOD loading rate of model parlor wastewater treated by activated sludge (control), (0.35ASM) for 0.35 BOD loading rate of model parlor wastewater treated by activated sludge containing *M. blollopis* SK-4, (0.52AS) for 0.52 BOD loading rate of model parlor wastewater treated by activated sludge (control) and (0.52ASM) for 0.52 BOD loading rate of model parlor wastewater treated by activated sludge containing *M. blollopis* SK-4. Abbrebiations for Figure 3 are biochemical oxygen demand (BOD), activated sludge (AS) and activated sludge containing *Mrakia blollopis* SK-4 (ASM).

Model wastewater containing cow's milk as a substrate of milk fat was prepared as an equivalent of BOD sludge loading in standard waste water treatment (0.35 kg-BOD/kg-MLSS·day). Activated sludge containing *M. blollopis* SK-4 had a BOD removal rate of 83.1%, higher than that in the control (63.8%, Fig. 2A). When BOD volume load was adjusted to 1.5 fold of standard wastewater treatment (0.52 kg-BOD/Kg-MLSS·day), BOD removal rate by activated sludge containing *M. blollopis* SK-4 was 80.1%, higher that in the control (65.2%, Fig. 2B). Regardless of BOD volume load, activated sludge containing *M. blollopis* SK-4 had a 1.25-fold higher BOD removal rate than that of the control.

### Production of lipase from Mrakia blollopis SK-4

Many microorganisms are known to produce lipase using Tween 80 as a substrate [18], [19]. Basidiomycetous yeast *M. blollopis* SK-4 also produced the enzyme. It is known that the production of lipase from *Candida rugosa* increased when yeast extract was used as a nitrogen source [20]. The same results as those for *M. blollopis* SK-4 were obtained. When 0.5% (w/v) yeast extract was used as a nitrogen source in the medium, lipase production by *M. blollopis* SK-4 lipase increased dramatically after 180 h. Maximum lipase activity of 0.695 U/mL was obtained with 0.5% (w/v) yeast extract, as compared to 0.059U/mL in its absence, after 324 h of inoculation, after which the accumulation of lipase markedly decreased (Spp. Fig. S1).

*M. blollopis* SK-4 morphology was observed by the fluorescence in situ hybridization (FISH) method during secretion of lipase. Therefore, all of the morphology of *M. blollopis* SK-4 during secretion of lipase was yeast form (data not shown).

### Purification of lipase

Lipase production medium was centrifuged at 3000×for 15 min at 4°C. The supernatant was filtered through a 0.45-μm membrane filter. The filtered solution was concentrated by ultrafiltration. After ultrafiltration, 72.3% of total lipase activities were recovered and 2.2-fold lipase specific activity was obtained. Then, enzyme solution was applied on a Toyopearl butyl-650 M column (2.5×20 cm) and purified by single-step hydrophobic interaction chromatography. Finally, 9.4% of the enzyme was recovered and 20.1-fold of specific activity, compared to crude sample, was obtained with a specific activity of 51.7 U/mg (Table 2). The purified enzyme showed a single band on SDS-PAGE with a molecular mass of 60 kDa (Supp. Fig. S2).

**Table 2:** Purification of lipase from *Mrakia blollopis* SK-4.

Purification step	Total protein (mg)	Total activity (U)	Specific activity (U/mg)	Recovery (%)	Fold
Supernatant of culture medium	108.0	278.0	2.5	100.0	1.0
Ultrafilter concentration	32.2	200.9	6.2	72.3	2.2
Toyopearl Butyl-650M	0.5	26.2	51.7	9.4	20.1

### Characterization of lipase

**Figure 3:**
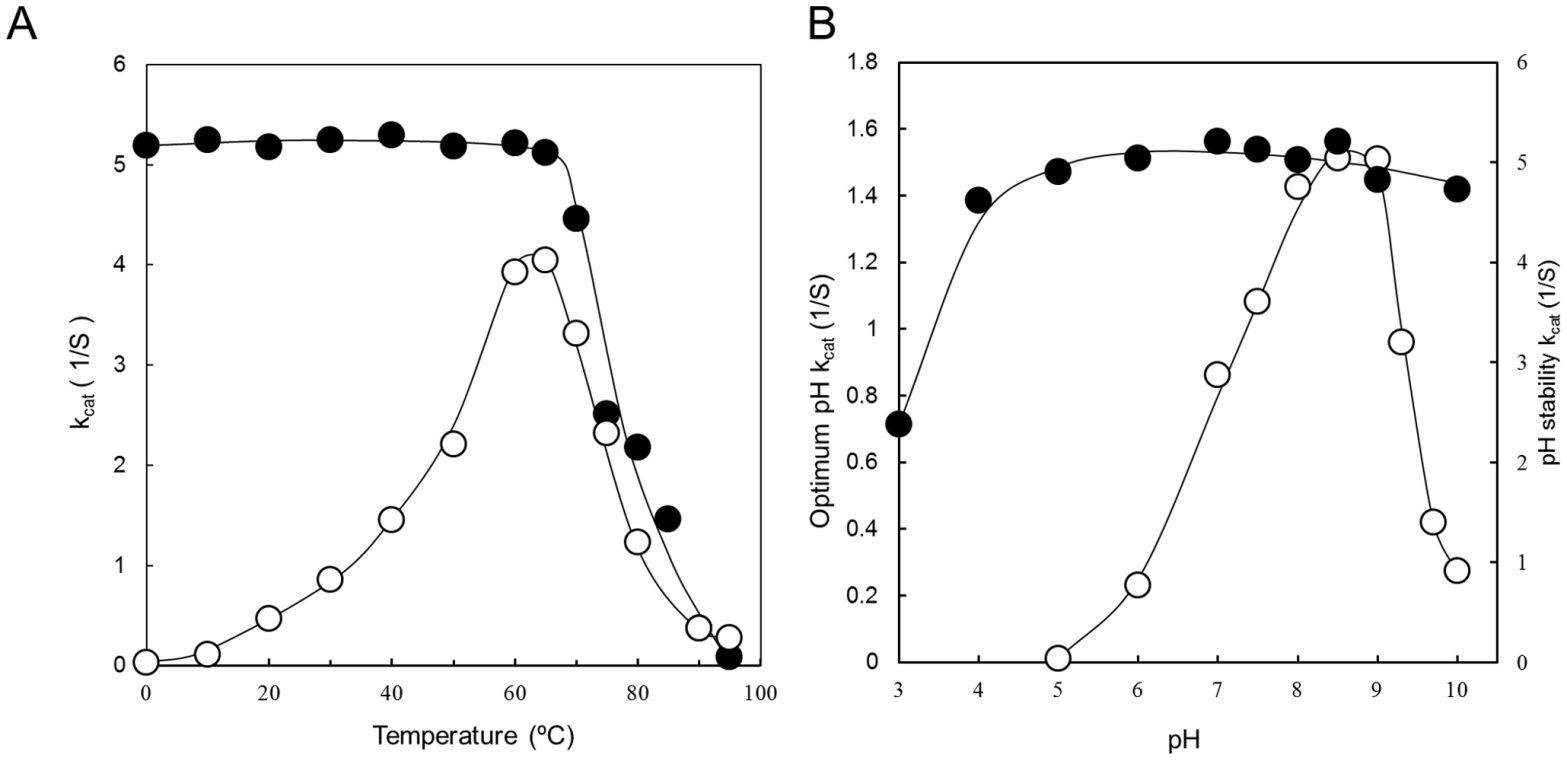
Effects of temperature and pH on SK-4 lipase. (A) Effects of temperature on lipase activity (○) and thermostability of the lipase (•). For effect of temperature, lipase activity assay was performed at various temperatures in 50 mM sodium phosphate buffer (pH 7.0) for 30 min. For thermostability, lipase was preincubated at various temperatures for 30 min. Remaining lipase activity was examined at 65°C for 30 min in 50 mM Tris-HCl buffer (pH 8.5). The line was fitted by using the Eyring-Arrhenius equation. (B) Effects of pH on lipase activity (○) and pH stability of lipase (•). For effect of pH, lipase activity assay was performed with various buffers at 30°C for 30 min using p-nitrophenyl-palmitate as a substrate. For pH stability, lipase was preincubated in various pH buffers at 30°C for 15 h and then pH of the buffer was adjusted to 8.5. The remaining enzyme activity was examined at 65°C for 30 min using p-nitrophenyl-palmitate as a substrate. The line was fitted by Henderson- Hasselbalch equation.

**Figure 4:**
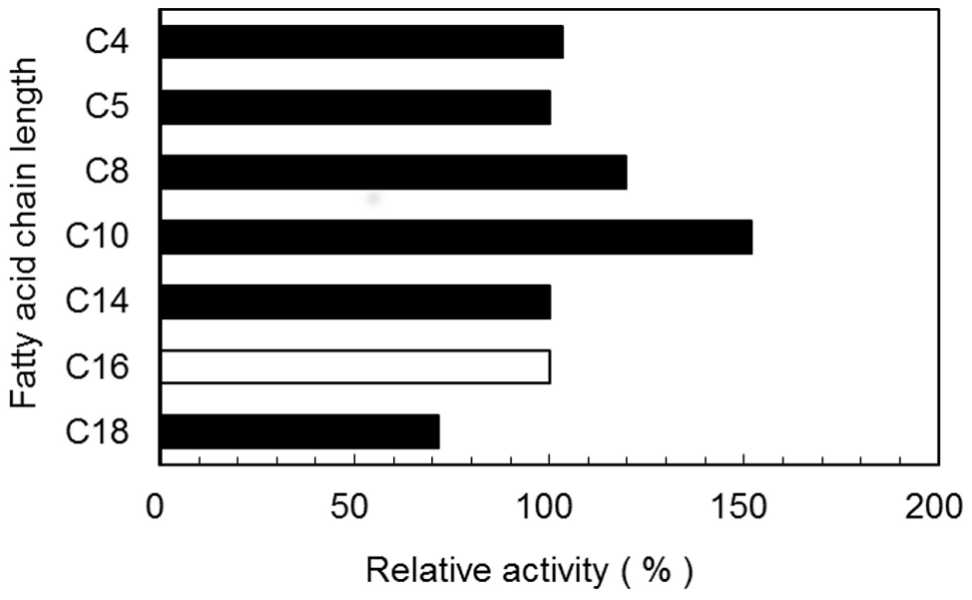
Substrate specificity of lipase form *Mrakia blollopis* SK-4. Substrates were p-nitrophenyl esters. Relative activity for each substrate (▪) is expressed as the percentage of activity toward p-nitrophenyl-palmitate (□).

Optimum temperature of lipase activity was 60−65°C (k_cat_  = 3.93 and 4.04 S^−1^). At temperatures of 80°C and 95°C, 30.5% (k_cat_  = 1.23 S^−1^) and 6.8% (k_cat_  = 0.27 S^−1^) of enzyme activity were retained (Fig. 3A). The enzyme showed thermo-stability up to 65°C with 98.3% (k_cat_  = 5.12 S^−1^) of residual enzyme activity even after 30-min preincubation. At 75°C, 80°C and 85°C, 48.2% (k_cat_  = 2.51 S^−1^), 41.8% (kcat  = 2.18 S^−1^) and 28.03% (k^cat^  = 1.46 S^−1^) of enzyme activity remained after 30-min preincubation (Fig. 3A). Optimum pH range of lipase activity was between pH 8.0 and pH 9.0 (kcat  = 1.43 and 1.51 S^−1^), whereas 57.0% (k_cat_  = 0.86 S^−1^), 63.5% (kcat  = 0.96 S^−1^) and 27.8% (kcat  = 0.42 S^−1^) of the enzyme activity was retained at pH 7.0, pH 9.3 and pH 9.7 compared to 100% at pH 8.5 (Fig. 3B). The enzyme was quite stable over a wide pH range (4.0−10.0) and 45.5% (k_cat_  = 2.38 S^−1^) of the enzyme activity remained at pH 3.0 even after preincubation for 15 h at 30°C (Fig. 3B). Enzyme activity was affected by metal ions at a concentration of 1 mM, retaining relative activity higher than 80%. There was little inhabitation of lipase activity in the presence of Cu^2+^ and Pb^2+^ ions. The metal-chelating agent EDTA did not affect lipase activity (Table 3). One of the most important characteristics of lipase was substrate specificity toward various p-nitrophenyl esters (C_4_−C_18_). The substrate specificity was determined in the presence of 1 mM p-nitrophenyl esters as a substrate with 50 mM sodium phosphate (pH 7.0) at 30°C for 30 min. Relative activity toward C_4_−C_14_ was more than 100% compared to 100% toward p-nitrophenyl-palmitate. C_18_ had relative activity of 71.6% (Fig. 4). Various organic solvents (ethanol, methanol, diethyl ether, dimethyl sulfoxide, hexane and *N, N*- dimethyl formamide) enhanced SK-4 lipase acidity. Solvents such as acetone and chloroform however, had only a slight inhibitory effect in the activity, with relative activities of 77.9% and 70.4%, respectively (Table 3). Organic solvents are known to be severely toxic to most enzymes including lipase [21].

**Table 3:** Comparison of the effects of various metal ions, EDTA and various organic solvents on *M. blollops* SK-4 and *Cryptococcus* sp. S-2 lipase activity.

Divalent cation or EDTA or Organic solvents	Relative activity (%)	
	*M. blollopis* SK-4	*Cryptococcus* sp. S-2
None	100.0	100.0
EDTA	106.7	ND
Metal salts		
MgCl_2_	102.2	>70
MnCl_2_	101.6	>70
FeCl_2_	90.0	>70
ZnCl_2_	83.6	ND
CoCl_2_	85.9	ND
CaCl_2_	112.8	>70
PbCl_2_	61.6	ND
CuCl_2_	67.2	<70
Ethanol	107.5	ND
Methanol	112.3	53.3
Diethyl ether	113.1	116.7
2– Propanol	95.5	ND
Dimethyl sulfoxide (DMSO)	134.3	121.4
Hexane	104.9	95.2
Acetone	77.9	83.3
Chloroform	70.4	74.8
*N, N*- dimethylformamide (DMF)	122.3	92.4

## Discussion

The Antarctic yeast strain SK-4, which we identified as *M. blollopis*, showed several uniques characteristics; for example, it can use various carbon sources as nutrition, it prefers relatively high temperature conditions among allied species and it can be activated even in vitamin-free conditions. These results suggested that strain SK-4 is a potential candidate for a biological agent to decompose various sugars in milk parlor wastewater under low temperature conditions. This expectation was further reconfirmed by the application of strain SK-4 in AS; namely, addition of SK-4 to AS in the model parlor wastewater improved the BOD removal rate.

Improved BOD removal rate of the activated sludge was attributed to lipase activity of SK-4 added. As expected, the lipase purified from *M. blollopis* SK-4 showed clearly weaker activity at lower temperatures but stronger activity in middle to high temperature conditions compared to activities of those from the other psychrophilic fungi [22]. *M. blollopis* SK-4 lipase, in addition, was quite stable in wide ranges of temperature and pH conditions and was not affected by the existence of EDTA, various metals ions, or organic solvents. *M. blollopis* SK-4 is thought to have acquired stable lipases by growing in extreme environments such as the Antarctica.

Comparison of the lipase from *M. blollopis* SK-4 with that from *Cryptococcus* sp. S-2, which has actually been used in wastewater treatment [23], [24], revealed that the former was superior to the latter both in thermo-stability and pH stability (Table 4); i.e., the lipase from *M. blollopis* SK-4 retained 71.1% of the enzyme activity after 30 min at 60°C and was stable for 6 hrs at 30°C in the pH range from 5.0 to 9.0.

**Table 4:** Comparison of *Mrakia blollopis* SK-4 lipase characteristics with other yeast lipase characteristics.

Yeasts	MW in kDa	Optimum pH	pH stability range	Optimum temperature
*Mrakia blollopis* SK-4	60	8.0∼9.0	4.0∼10.0	60–65°C
*Candida antarctaca*	43 (lipaseA)	7.0	-	50°C
*Candida rugosa* ATCC1483	60	5.0	-	-
*Cryptococcus* sp. S-2	22	7.0	5.0-9.0	37°C
*Geotrichum candidum*	55	5.6∼7.0	4.2∼9.8	40°C
*Yarrowia lipolytica*	44 (lip)	8.0 (lip)	4.5∼8.0	37°C
*Kurtzmanomyces* sp. L-11	49	1.9∼7.2	Below 7.1	75°C
*Trichosporon asteroids*	37	5.0	3.0∼10.0	50°C

In conclusion, *M. blollopis* SK-4 has the ability to assimilate various carbon compounds and to use various sugars for fermentation. Moreover, the lipase is more tolerant in relatively higher temperature conditions and wider pH ranges, less sensitive to various metal ions and organic solvents, and highly reactive to various chain lengths of substrates. SK-4 lipase, therefore, is a promising biological agent for parlor wastewater treatment even in low temperature regions of the world.

## Supporting Information

Figure S1 **Effect of yeast extract on lipase production.** *Mrakia blollopis* SK-4 was cultivated by lipase production medium (♦) and lipase production medium without yeast extract (•).
(TIF)

Figure S2 **SDS-PAGE of purified lipase from Mrakia blollopis SK-4.** Lane 1. Molecular weight marker; 2. Purified lipase.
(TIF)
