# Supplementary figures and images for: An Application of Wastewater Treatment in a Cold Environment and Stable Lipase Production of Antarctic Basidiomycetous Yeast Mrakia blollopis
Source: PLoS One. 2013 Mar 14;8(3):e59376. doi: 10.1371/journal.pone.0059376 (PMC3597603; doi:10.1371/journal.pone.0059376)

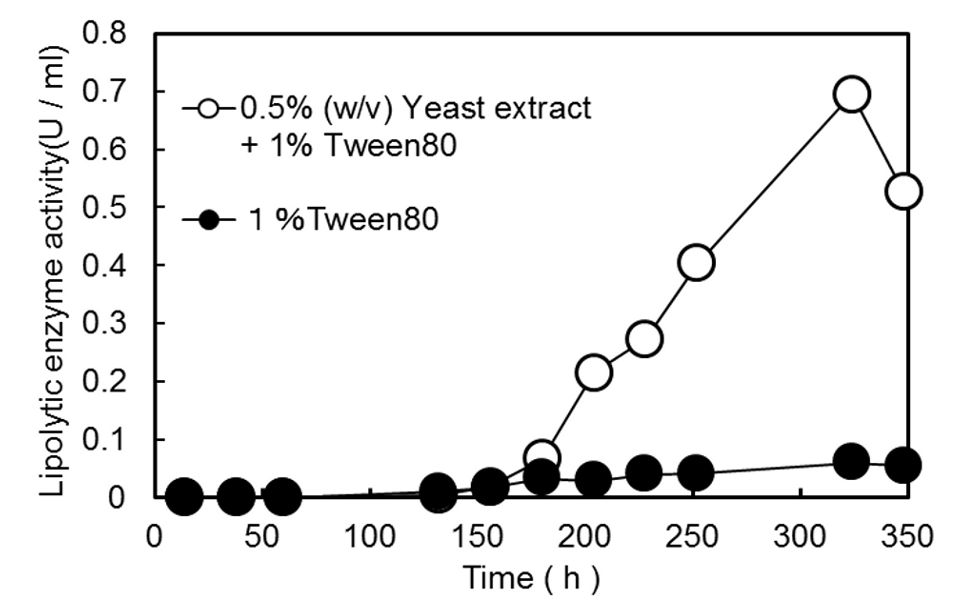

Supplement: Figure S1 — Effect of yeast extract on lipase production. Mrakia blollopis SK-4 was cultivated by lipase production medium (♦) and lipase production medium without yeast extract (•). (TIF) [file pone.0059376.s001.tif]

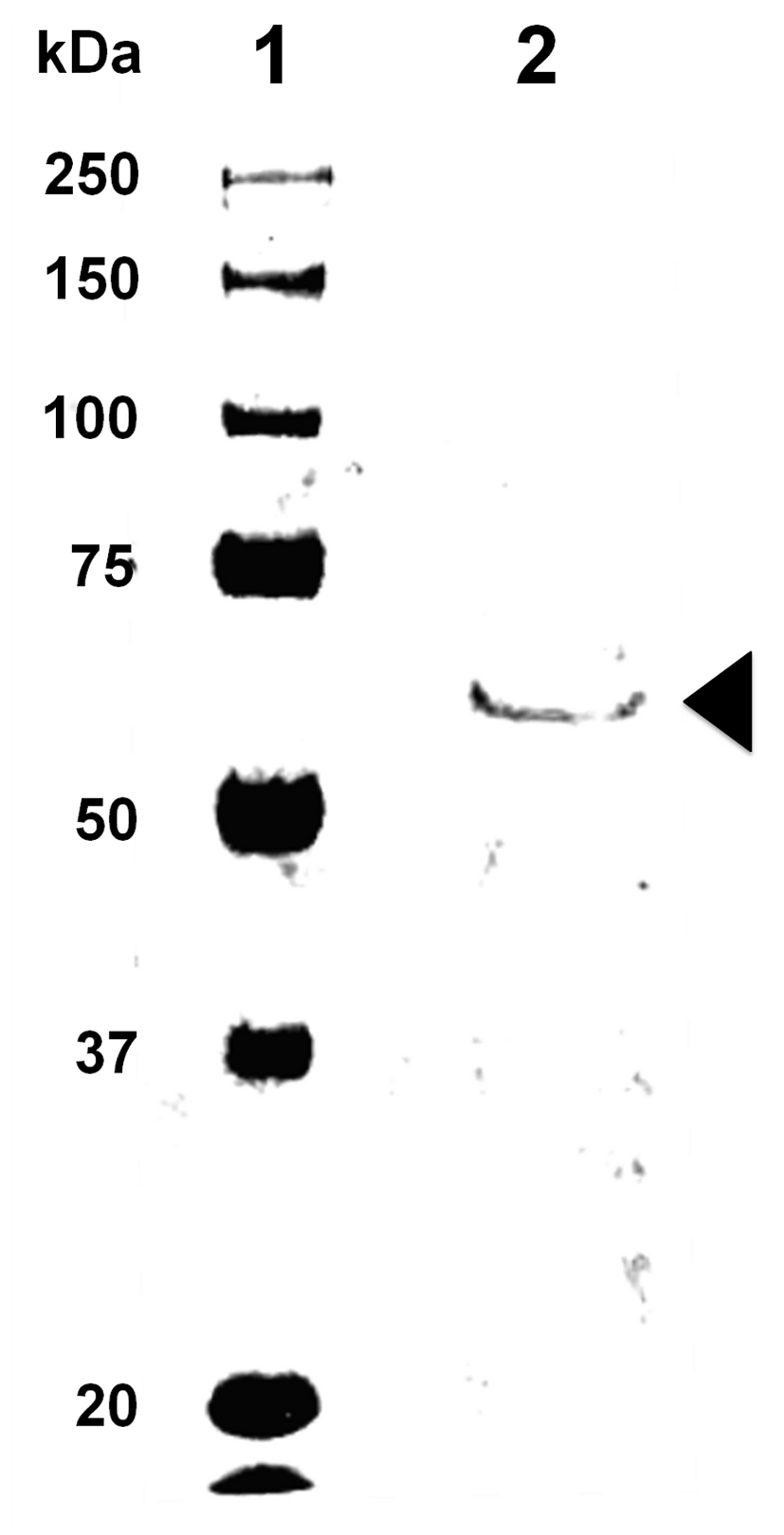

Supplement: Figure S2 — SDS-PAGE of purified lipase from Mrakia blollopis SK-4. Lane 1. Molecular weight marker; 2. Purified lipase. (TIF) [file pone.0059376.s002.tif]
